# Meta-Analysis of Human IgG anti-HEV Seroprevalence in Industrialized Countries and a Review of Literature

**DOI:** 10.3390/v11010084

**Published:** 2019-01-20

**Authors:** Lisandru Capai, Alessandra Falchi, Rémi Charrel

**Affiliations:** 1 EA7310 BIOSCOPE, Laboratoire de Virologie, Université de Corse-Inserm, 20250 Corte, France; 2 Unité des Virus Emergents (UVE), Aix-Marseille Université, IRD 190, INSERM 1207, IHU Méditerranée Infection, 13005 Marseille, France; remi.charrel@univ-amu.fr; 3 Emerging Pathogens Institute, University of Florida, Gainesville, FL 32611, USA

**Keywords:** epidemiology, Hepatitis E virus, Meta-analysis, seroprevalence, industrialized countries

## Abstract

Although Hepatitis E is increasingly described as a major cause of liver disease in industrialized countries, the epidemiology is far from being fully elucidated. We provide here a comprehensive review of documented clusters of cases, and of serological studies conducted in populations with distinct types of exposure. Seroprevalence rates range from <5% to >50% depending on the countries and the groups of population. Such discrepancies can be attributed to the type of serological assay used, but this solves only a part of the problem. We performed a meta-analysis of studies performed with the broadly used Wantai HEV-IgG ELISA and found striking differences that remain difficult to understand with the current knowledge of transmission pathways.

## 1. Introduction

Hepatitis E virus (HEV) is the major cause of enterically transmitted hepatitis worldwide [1]. There are two contrasting situations around the world with regard to hepatitis E. On the one hand, in developing countries, transmission is mainly fecal-oral with poorly treated water playing a major role. In these countries, the incriminated genotypes are HEV-1 and HEV-2. On the other hand, in industrialized countries, although not all transmission pathways are well known [2], the main form of transmission is zoonotic with HEV-3 and HEV-4 genotypes [3,4]. A growing number of studies have been published concerning the seroprevalence of HEV antibodies in different human populations. Hyperendemic zones also appear in industrialized countries with seroprevalences of up to 70% in some regions. However, considerable heterogeneity exists depending upon either the type of method used or the population under study, or both. In high-income countries, where swine are the main reservoir, very high IgG anti-HEV seropositivity rates can be observed.

HEV is a small, single-stranded, positive-sense, RNA virus (~7.2 kb) [5]. HEV was initially known as a nonenveloped virus; however, another form has recently been identified. This form can be “masked” by the membrane of the host cells and is resistant to antibodies when it is in the blood [6,7]. When HEV particles are released by the cellular exosomal pathway, it is considered a “quasi-enveloped” virus. It contains genome coding for three open reading frames (ORF1, ORF2, and ORF3). In HEV genotype 1, a fourth ORF was discovered on ORF1, namely ORF4 [8]. HEV belongs to the Hepeviridae family [3]. Since 2015, this family has been divided into two genera, Orthohepevirus and Piscihepevirus [9,10]. Orthohepevirus contains four species named Orthohepevirus A to D. Currently, all strains isolated from humans belong to Orthohepevirus A in these five genotypes: HEV-1, HEV-2, HEV-3, HEV-4, and HEV-7. Genotypes 1 and 2 only affect humans but other genotypes can also infect other mammals. Orthohepevirus B, C, and D consist of avian, rat/carnivore, and bat viruses, respectively. The genus Piscihepevirus includes a single species (A) whose typical isolate, cutthroat trout virus, infects trout, although its pathogenicity and full host range are unknown [11].

The balance between symptomatic and asymptomatic forms is not yet well known. Certainly, with the very high seroprevalence observed in certain “hyperendemic” regions and the number of clinical cases recorded, infection by HEV is predominantly asymptomatic [12,13,14]. However, there are different forms of clinical manifestations that can range from acute hepatitis to fulminant hepatitis (1–2% of cases) [15]. Extrahepatic forms may also exist, including neurological disorders, thrombocytopenia, kidney injury, hemolytic anemia, and pancreatitis [16,17,18]. Chronic infections have been reported in most cases with genotype 3 infection in immunocompromised people in industrialized countries [19,20,21,22,23,24,25]. Recently, new chronic infections have been found to have been caused by genotype 4 [1,26,27,28,29,30,31].

Our understanding of the epidemiology of HEV in industrialized countries for human populations is complicated given the large number of studies, the heterogeneity of the laboratory tests used, and the different types of population included. The purpose of this review is to summarize the situation in industrialized countries with regard to humans. We review the recent detection of HEV markers and grouped cases among humans. Furthermore, a meta-analysis was performed to determine the overall seroprevalence of anti-HEV IgG in the general population in industrialized countries.

## 2. Seroprevalence of IgG anti-HEV

Anti-HEV IgG antibodies represent a marker of previous exposure to HEV. However, wide variations in sensitivity and specificity rates of various anti-HEV IgG assays make the interpretation of seroepidemiological studies of HEV infection difficult [32,33,34].

### 2.1. Seroprevalence of IgG anti-HEV with Wantai HEV-IgG ELISA Assay

Currently, the Wantai assay is the most commonly used assay with specificity and sensitivity for the HEV IgG of 97.96% and 99.6%, respectively [35,36]. In addition, other studies showed a better detection rate with the Wantai assay than other assays and this difference was probably not due to false positives [37,38]. Indeed, Wantai assay shows very low seroprevalences in children or some countries and increasing values with age [39,40]. Moreover, the main part of positives samples was confirmed by immunoblot [37].

As shown in Table 1, the non-Wantai assays underestimate HEV seroprevalence. A Danish study estimated seroprevalences of 10.7% and 19.8% for HEV antibodies depending on the assay used for NIH and Wantai, respectively [41]. The prevalence of IgG anti-HEV in Catalan blood donors was 19.96% (Wantai assay) and 10.72% (Mikrogen assay) [42]. We can also compare the use of different assays in different studies of the same population. Two studies in the Czech Republic, using different assays, found seroprevalences in BD of 5.7% and 6.7% [43,44]. Although the percentages here are similar, this is not always the case. The seroprevalence rate in France ranges from 3.2% to 22.4% depending on the type of detection assay (Genelabs Diagnostics vs. Wantai) [45,46].

Of the 5 studies which have tested the same sera using at least 2 different methods, the Wantai IgG kit was used on 4 occasions: in all of these 4 cases, the prevalence of IgG observed with the Wantai IgG kit was higher than that obtained with other kits [41,42,43,47,48].

### 2.2. Meta-Analysis

A meta-analysis was carried out in accordance with the “Preferred Reporting Items for Systematic Reviews and Meta-Analyses” (PRISMA) guide [49].

#### 2.2.1. Literature Search, Selection Criteria, and Study Quality

A literature search was made in PubMed using the keywords “hepatitis E seroprevalence” or “hepatitis E blood donors”.

**Figure 1:**
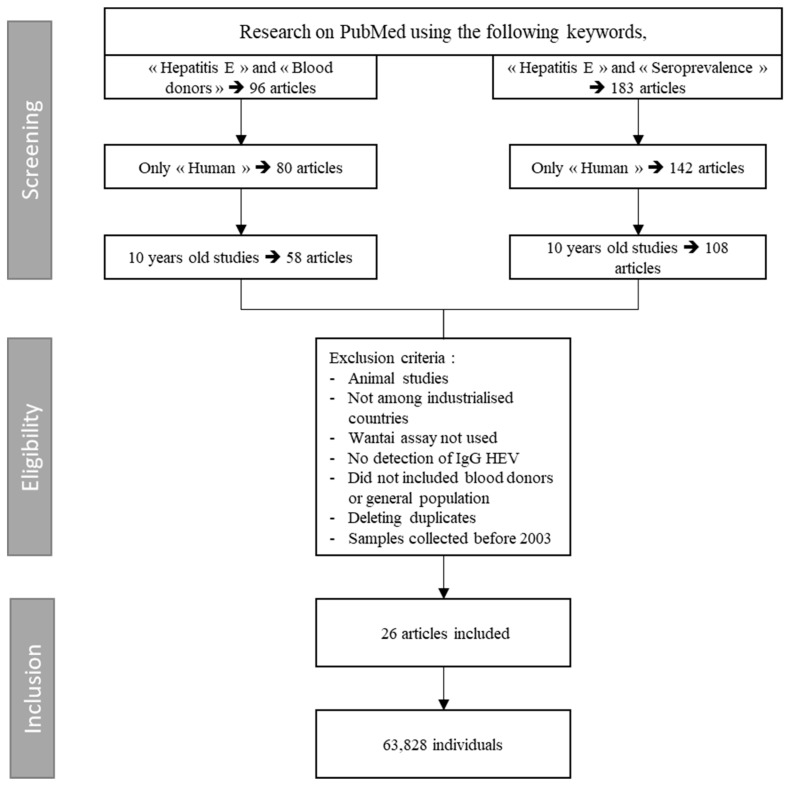
Search algorithm for meta-analysis: HEV IgG seroprevalence among blood donors.

Selection was based on title and abstract, and excluded studies older than 10 years and those where samples were collected before 2003, reviews, animal studies, and studies with a sample size smaller than 20 individuals or sera. The populations retained were the general population and the blood donors. No language restrictions were applied. Only studies regarding seroprevalence rates in industrialized countries with Wantai assay were included. Original abstracts were obtained and assessed in detail for inclusion. Following the abstract review, the full papers of the included studies were reviewed (Figure 1).

#### 2.2.2. Data Extraction

Information was extracted from each study included authors, journal, year of publication, country, diagnostic assay used, number of patients, positive events, seroprevalence, and kind of patient cohort. Data were stratified for three variables: assay employed, country of study, and nature of study cohort. We focused on different study cohorts: general population/blood donors, immunocompromised patients, and individuals with contact with swine/wild animals. The latter included veterinarians, farmers, hunters, forestry workers, and pig farm workers.

#### 2.2.3. Statistical Analysis

The overall seroprevalence of anti-HEV IgG in industrialized countries was estimated by pooling the study data to run a meta-analysis. The analysis was conducted using the R statistical platform (version 3.1.2) and the metafor package (version 1.9-5) [50]. The I2 statistic was used to estimate the amount of heterogeneity accounted for by each model [51]. We used the double arcsine transformation method for variance stabilization [52] and a restricted maximum likelihood (REML) estimator for prevalence estimation.

#### 2.2.4. Results of Meta-Analysis: Human Seroprevalence of IgG anti-HEV

**Figure 2:**
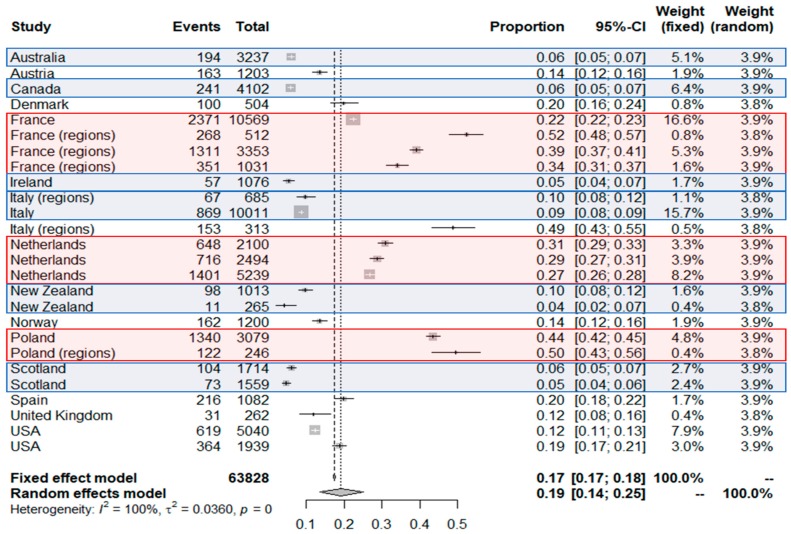
Results of the meta-analysis: Seroprevalence among blood donors in industrialized countries using Wantai HEV-IgG ELISA assay. Red areas correspond to countries with seroprevalence higher than the overall seroprevalence and blue areas to those with lower value. Black crosses correspond to the seroprevalence value of each study. The size of the grey square around the cross depend on the number of samples of each study included (weight). Black bars around the point correspond to the confidential interval. The additional data for each study included in the meta-analysis are presented in Table 2.

A total of 26 studies from 15 countries were included in the meta-analysis of seroprevalence in industrialized countries using Wantai assays. The overall seroprevalence of IgG anti-HEV calculated was 19% [14–25%] and the I2 showed high heterogeneity (100%) (Figure 2). Given the heterogeneity between studies, the random effects model was chosen. Reported seroprevalences ranged from 4.2% to 52.5% [40,53]. The 26 studies comprised a total of 63,828 individuals. The list of references associated with the studies of the meta-analysis is present in the Table 2.

#### 2.2.5. Interpretation of Meta-Analysis

France, Poland, and the Netherlands appear as countries with the highest seroprevalences. France has a fairly average national seroprevalence but regions of hyperendemias appear. For the Netherlands and Poland, the data of the different studies are homogeneous around 30% and 50% respectively. New Zealand, Scotland, Australia, Canada, and Ireland have seroprevalence under 10%. A special case appears with studies in Italy. In fact, for this country very distant seroprevalences have been observed, and hyperendemic zones could also exist in this country. Interestingly, in the 6 countries where at least 2 studies were done, the order of magnitude is well observed, specifically in the Netherlands (0.27; 0.29; 0.31), in Poland (0.44; 0.50), in Scotland (0.05; 0.06), in New Zealand (0.04; 0.1); in France, rates at 0.22 (national), 0.34 (Southwest France), 0.39 and 0.52 (Southern France) were observed; in Italy, the 3 studies provided very different rates at around 0.09 (national and Northern Italy) and 0.49 (Central Italy): These high differences in some regions may be explained by local eating habits or environmental HEV contamination.

### 2.3. High-Exposure Populations

People working in contact with animals (forest workers, hunters, pig and other farm workers) appeared as categories with higher seroprevalences (Table 3). It is impossible to compare studies that have not used the same assays. On the other hand, some studies have compared, under the same conditions, a GP with an “exposed population” (range 13.4–31.2%) [73,74]. While these studies were not conducted with the Wantai HEV-IgG ELISA assay, most of them were performed compared with a reference population. Accordingly, it can be deduced that selected populations are at higher risk of HEV infection such as pig farm workers or human with occupational exposure to pigs [73,75,76,77,78], forest workers [74,78,79], and farm workers [63]. Interestingly, veterinarians do not appear always as having a higher risk of infection [63,80,81]. However, the type of veterinarian activity (rural or urban) may greatly influence the risk of exposure to HEV as shown in Norway where seroprevalence in veterinarians working with swine was two times higher compared to those who did not work with swine [63].

### 2.4. Children

The few studies conducted in children consistently reported low seroprevalence rates: <5% in the USA, 4.2% in Turkey, 4.6% in Spain, 1.4% in Russia and 1.1% in Portugal [85,86,87,88,89] (Table 4).

### 2.5. Other Seroprevalences Observed in Industrialized Countries

Several studies have determined HEV seroprevalence through using assays other that the Wantai HEV-IgG ELISA. Seropositivity rates ranged between 1.9% in the Netherlands and 16.8% in Germany (Mikrogen) (Table 5) [90,91]. However, it is difficult to interpret these results and to compare results obtained by using different kits as previously indicated. These values may be useful whether the same kit is used in future studies or to compare exposure in different countries, in different populations or at different time points provided the same technique is used. These results should be considered as reference values for further studies performed using the same kit.

### 2.6. Characteristics Associated with Higher Seroprevalence

The main characteristics associated with a high seropositivity rate in industrialized countries are increasing age, sex (male), contact with animals, consumption of raw or uncooked pork liver sausages (including ficatelli, a traditional Corsican pork liver sausage and traditional Dutch dry raw sausages), frequent consumption of bovine steak, frequent consumption of smoked beef offal, and consumption of oysters [45,59]. Conversely, bottled drinking water was associated with lower levels of anti-HEV IgG [45]. However, other factors could influence the exposure of the population to the virus.

## 3. Outbreaks in High-Income Countries

### 3.1. Confirmed and Grouped Cases

The European Centre for Disease Prevention and Control (ECDC) reported that the number of confirmed HEV cases had increased from 514 in 2005 to 5617 in 2015, with 21,018 total reported cases during the whole period [110]. Three countries (France, the UK, and Germany) accounted for 80% of all reported cases. The number of severe cases also increased between 2005 (*n* = 85) and 2015 (*n* = 1115). The sex ratio (male) ranged from 61% to 69%. The proportion of cases in people older than 50 increased from ~40% during the 2005–2008 period to >60% during the 2013–2015 period. During the 2005–2015 period, a total of 28 deaths attributable to HEV have been claimed, with a clear increase since 2009.

Although HEV epidemics are predominantly due to genotypes 1 and 2 in tropical countries, small outbreaks have been described in industrialized countries. The ECDC reported 37 outbreaks between 2005 and 2015 with an increasing trend after 2009 [111]. The size of the outbreaks reported is ranged from 2 to 47 cases per year. Data on HEV outbreaks are provided by 18 countries and among them, 11 did not report any outbreak.

### 3.2. Description of Outbreaks

Table 6 summarizes seven HEV outbreaks in industrialized countries that involved 4–17 cases. Two were caused by HEV genotype 3 with the common source of contamination being undercooked pig or wild boar.

In Spain, in October 2015, an HIV-infected patient presented at the Infectious Diseases Unit of the Hospital Universitario Reina Sofía de Córdoba with malaise, diarrhea, jaundice, vomiting, and fever. He tested positive for the presence of HEV RNA in her serum. The epidemiological investigation concluded that (i) all 8 family members had detectable HEV RNA in the serum, (ii) they were infected through the consumption of wild boar meat as demonstrated by HEV RNA detection in remaining frozen meat collected at home, (iii) genetic analysis showed that the 8 family members and the remaining frozen meat corresponded to the same viral strain (100% identity) belonging to genotype 3 [112]. This sequence was previously described in wild boars from south-central Spain and swine (GenBank accession number: EU723512).

In France, in 2013, a cluster of 17 cases was associated with consumption of a spit-roasted piglet during a wedding [113]. Twelve cases (70%) were non symptomatic, five were symptomatic, and among them two (11.7%) were hospitalized. All individuals recovered. The survey attributed this large HEV outbreak to the consumption of an undercooked pig liver-based stuffing.

In the UK, in 2008, four passengers on a cruise ship presented with HEV infection [14]. Because of the incubation period, the source of infection was not identified.

In Italy, in 2011, five persons living in the Lazio region (close to Roma) were infected by HEV [114]. Despite the source of infection not being identified, the causing strain belonged to genotype 4 and was genetically most closely related with strains from China; none of these persons had contact or consumption history of Chinese products. These results are in line with those provided by other studies indicating that genotype 4 is now established in Europe.

The Czech Republic was also affected by two outbreaks [115]. For the first 13-case outbreak, the source was not identified. In the second outbreak, a link between human cases and infection in farm pigs was revealed for the first time.

In Australia, 55 cases of HEV infection were confirmed, including 24 people who had not traveled during the incubation period [116]. Of these, 17 had eaten in the same restaurant. The HEV RNA detected was of genotype 3. The high attack rates and OR estimated in the study were for pork products.

In 2016, after the notification of hepatitis E cases to the Asahikawa City Center of Public Health (ACPH) in a nursing home for elderly in Hokkaido, Japan, 125 persons were tested for HEV infection markers, and 29 were found to be positive. Only four presented anorexia, fatigue, and abdominal pain. All cases were caused by strains belonging to genotype 3 [117] and amplified sequences presented 99.8 to 100% of homology.

## 4. Conclusions

The markers of HEV infection are found at an increasing frequency among humans in industrialized countries. In addition, the sporadic nature of human cases in these countries is no longer relevant; hyperendemic regions exist in France, Italy, and Poland, at least. The number of confirmed cases and of clusters of cases has also drastically increased during the last decade. Whether this is due to a true epidemiological trend or due to increased awareness to HEV is not clear. The meta-analysis of studies performed with the Wantai HEV-IgG ELISA on blood donors or healthy general populations allowed us to classify industrialized countries into one of the following 3 categories based on the 19% (CI95 14–26) cut-off prevalence rate: high risk (France, Netherlands, Poland), medium risk (Austria, Denmark, Norway, Spain, UK and USA), and low risk (Canada, Scotland, Ireland, Australia and New Zealand). In addition, there is clearly an occupational risk for forest workers, farm workers, and pig farm workers. Apart from the pork-eating route of infection, the high exposure of adult population in industrialized countries (lowest rate is 5%) demands future investigations to better understand how people acquire HEV infection. The great heterogeneity observed between the anti-HEV IgG detection assays makes the comparison of the different studies and the comprehension of HEV epidemiology difficult. The establishment of a reference method in seroprevalence studies could remedy this problem.

## Figures and Tables

**Table 1:** HEV IgG prevalences observed when the same population is tested using different assays.

Country	Population	Assay	Number of Samples	Seroprevalence anti-HEV IgG (%)	Reference
Denmark	BD	Wantai	504	19.80	[41]
		NIH		10.70	
Spain	BD	Wantai	9998	19.96	[42]
		Mikrogen		10.72	
Czech Republic	GP	Mikrogen	1715	6.70	[43]
		Diagnostic Bioprobes	230	5.70	[44]
France	BD	Wantai	10,569	22.4	[45]
		Genelabs Diagnostics	1998	3.20	[46]
New Zealand	BD	Wantai	1013	9.70	[47]
		MP Diagnostics		8.10	
USA	BD	Wantai	5040	12.28	[48]
		DSI		11.29	
		MP Diagnostics		10.65	

BD, blood donors; GP, general population. Lists of assays: Wantai: HEV-IgG ELISA; NIH, in-house assay developed at the National Institutes of Health, Mikrogen: RecomLine Anti-HEV IgG; Diagnostic Bioprobes: ELISA kit HEV; Genelabs diagnostics: HEV ELISA; MP Diagnostic: HEV ELISA; DSI, Diagnostic Systems Incorporated.: DS-EIA-ANTI-HEV-G.

**Table 2:** Comparison of seroprevalence rates observed with the Wantai HEV-IgG ELISA assay in blood donors (BD) and the general population (GP) (data used for meta-analyses). “Country (regions)” means the seroprevalence is at regional level.

Country	Population	Number of Samples	Positive Samples	Seroprevalence anti-HEV IgG (%)	Reference
France (regions)	BD	512	268	52.5	[40]
Poland (regions)	BD	246	122	49.6	[54]
Italy (regions)	BD	313	153	49	[55]
Poland	BD	3079	1340	43.52	[56]
France (regions)	BD	3353	1311	39.1	[57]
France (regions)	BD	1031	351	34	[58]
Netherlands	BD	2100	648	31	[59]
	GP	2494	716	28.7	[60]
	BD	5239	1401	27	[61]
France	BD	10,569	2371	22.4	[45]
Spain	BD	1082	216	19.96	[42]
Denmark	BD	504	100	19.8	[41]
USA	BD	1939	364	18.8	[62]
Norway	BD	1200	162	14	[63]
Austria	BD	1203	163	13.55	[64]
USA	BD	5040	619	12.28	[48]
UK	BD	262	31	12	[65]
Italy (regions)	BD	685	67	9.8	[66]
New Zealand	BD	1013	98	9.7	[47]
Italy	BD	10,011	869	8.7	[67]
Scotland	BD	1714	104	6.1	[68]
Australia	BD	3237	194	5.99	[69]
Canada	BD	4102	241	5.9	[70]
Ireland	BD	1076	57	5.3	[71]
Scotland	BD	1559	73	4.7	[72]
New Zealand	BD	265	11	4.2	[53]

**Table 3:** HEV IgG rates observed in different populations.

Country	Population	Assay	Number of Samples	Seroprevalence anti-HEV IgG (%)	*p*	OR [CI95]	References
Estonia	H	Mikrogen	144	4.2	0.02	3.54 [1.07; 12.69]	[73]
	PFW		67	13.4			
Germany	BD	Mikrogen	301	11	0.0076	1.77 [1.15; 2.794]	[79]
	FW		563	18			
	GP		106	28.3	0.02	2.14 [1.06; 4.41]	[82]
	PFW		116	15.5			
Norway	BD	Wantai	1200	14	*NS*, *p* > 0.05		[63]
	V		163	13			
	FaW		79	30	0.00028	2.68 [1.54; 4.53]	
Portugal	GP	Mikrogen	804	19.9	0.01	1.78 [1.12; 2.79]	[76]
	PFW		114	30.4			
Spain	GP	ND	325	7.3	0.002	2.89 [1.42; 5.82]	[83]
	PFW		101	19			
Finland	GP	Axiom	52	5.8	*NS*, *p* > 0.05		[80]
	V		333	10.2			
USA	BD	In-house assay	400	18	0.005	1.60 [1.14; 2.26]	[77]
	PFW		468	26			
France	FW	MP Diagnostics	593	31.2	/	/	[74]
	GP		322	26	0.007	1.61 [1.13; 2.30]	[78]
	FW		306	36.4			
	PFW		231	43.8	0.0000017	2.19 [1.51; 3.21]	
Italy	BD	BioChain Institute	170	8.82	0.0007	3.30 [1.53; 8.15]	[81]
	V		83	9.64			
Poland	H	ND	210	3.81	/	/	[84]

H: Hunters; PFW: Pig farm workers or with occupational contact with pigs; BD: blood donors; FW: Forest workers; FaW: Farmworkers; V: Veterinarians; GP: General population. Lists of assays: Mikrogen: RecomLine Anti-HEV IgG; Axiom: HEV IgG EIA; MP Diagnostic: HEV ELISA; Biochain: ELISA Kit for IgG Antibody to Hepatitis E Virus ND: assay not determined.

**Table 4:** HEV IgG prevalence observed in children.

Country	Population	Assay	Number of Samples	Seroprevalence anti-HEV IgG (%)	Reference
USA	CH	ND	18,695	<5	[85]
Spain	CH	Biokit	1249	4.6	[86]
Turkey	CH	Diagnostic Bioprobes	408	4.2	[87]
Russia	CH	ND	3122	1.4	[88]
Portugal	CH	Mikrogen	352	1.1	[89]

CH: children. Lists of assays: Biokit: bioelisa HEV IgM 3.0; Mikrogen: RecomLine Anti-HEV IgG; Euroimmun: Anti IgG HEv ELISA; Diagnostic Bioprobes: HEV IgG. ND: not determined assay.

**Table 5:** Prevalences of HEV IgG observed in industrialized countries with assays other that Wantai HEV-IgG ELISA.

Country	Population	Assay	Number of Samples	HEV IgG (%)	Reference
Belgium	P	Strip immunoassay	100	14	[92]
Germany	GP	Mikrogen	4422	16.8	[90]
Germany	GP		7075	15.3	[93]
Greece	BD	Adaltis	265	9.43	[94]
Hungary	P	In-house assay	246	10.5	[95]
Ireland	BD	ND	198	8	[96]
Israel	BD	Diagnostic system	729	10.6	[97]
Italy	BD	In-house assay	199	7	[98]
Japan	BD	In-house assay	12,600	3.4	[99]
Japan	GP	In-house assay	22,027	5.3	[100]
Netherlands	GP	ND	7072	1.9	[91]
Poland	BD	Euroimmun	105	3.8	[101]
Portugal	BD	Abbott	1473	2.5	[102]
Spain	BD		563	2.8	[103]
Spain	GP	ND	NA	10	[104]
Spain	GP	Bioprobes	2305	2.17	[105]
Switzerland	BD	MP Diagnostics	550	4.9	[106]
UK	BD	ND	500	16	[107]
UK	PCLD	ND	126	13	
UK	OP	ND	336	25	
USA	GP	Diagnostic system	5966	10.2	[108]
USA	GP		7885	6	
USA	BD	MP Diagnostics	4499	9.5	[109]

BD: blood donors; PS: Professional soldiers; P: Patients; GP: General population; H: Hunters; PFW: Pig farm workers or in contact with pigs during job; FW: Forest workers; CH: Children; V: Veterinarians; FaW: Farmworkers; PCLD: Patients with chronic liver disease; OP: Old people; ND: assay not determined. Lists of assays: Mikrogen: RecomLine Anti-HEV IgG; Adaltis: AIAgen HEV; Diagnostic system: Anti-HEV-IgG kit; Euroimmun: Anti IgG HEv ELISA; Bioprobes: HEV IgG Diagnostic; MP Diagnostic: HEV ELISA. Abbott: HEV EIA.

**Table 6:** List of HEV outbreaks for which peer-reviewed data are available.

Country	Number of Persons	Year	Cause	Virus	Reference
Spain	8	2015	Wild boar meat consumption	Genotype 3	[112]
Australia	17	2014	Pork products in a restaurant	Genotype 3	[116]
France	17	2013	Wedding spit-roasted piglet	ND	[113]
Italy	5	2011	Not identified	Genotype 4	[114]
Czech Republic	13	Not found in the reference	Not identified	ND	[115]
	8		Infection in farm pigs	ND	
UK	4	2008	foodborne source on a cruise ship	ND	[14]
Japan	29	2016	Not identified	Genotype 3	[117]

ND: Not determined.
